# Double-Blinding and Bias in Medication and Cognitive-Behavioral Therapy Trials for Major Depressive Disorder

**DOI:** 10.12688/f1000research.6953.2

**Published:** 2016-02-04

**Authors:** Douglas Berger

**Affiliations:** 1 Meguro Counseling Center, Tokyo, Japan

**Keywords:** psychotherapy, cognitive-behavioral therapy, outcome studies, blinding, clinical trials

## Abstract

While double-blinding is a crucial aspect of study design in an interventional clinical trial of medication for a disorder with subjective endpoints such as major depressive disorder, psychotherapy clinical trials, particularly cognitive-behavioral therapy trials, cannot be double-blinded. This paper highlights the evidence-based medicine problem of double-blinding in the outcome research of a psychotherapy and opines that psychotherapy clinical trials should be called, “partially-controlled clinical data” because they are not double-blinded. The implications for practice are, 1. For practitioners to be clear with patients the level of rigor to which interventions have been studied, 2. For authors of psychotherapy outcome studies to be clear that the problem in the inability to blind a psychotherapy trial severely restricts the validity of any conclusions that can be drawn, and 3. To petition National Health Insurance plans to use caution in approving interventions studied without double-blinded confirmatory trials as they may lead patients to avoid other treatments shown to be effective in double-blinded trials.

Psychotherapy clinical outcome trials for major depressive disorder (MDD) are often described as “randomized”, “controlled”, “single-blind”, etc. These words may not adequately describe the level of methodologic rigor of the design of a trial for MDD because the endpoints are subjective symptom ratings ^1^, and the inability to double-blind MDD psychotherapy outcome trials is a crucial problem in the methodology of these trials ^2^. Cognitive-behavioral therapy (CBT) is a widely-used type of psychotherapy in the treatment of MDD, however, CBT is very difficult if not impossible to double-blind because the subjects are actively involved in the therapy ^3^. While clinical trials of CBT are often called “single-blind” because the raters are blind to treatment allocation, “single-blind” in a clinical trial is actually defined as a case where the subjects are blind, not the raters ^2, 4^.

The evaluation of MDD efficacy is more complex in some ways than that for objective endpoints of, say, tumor size, cholesterol level, or survival years. MDD may be diagnosed in a variety of persons, some with more psychological distress, and some with more neurovegetative symptoms. The symptoms of MDD are measured on rating scales whose scores become the endpoints of the study. All of the items on these rating scales are subjective, and some items like hopelessness and low self-esteem are likely to improve with non-specific aspects of receiving care that include the hope and expectation inherent in belief in the treatment, compared with other symptoms such as lethargy and insomnia ^5^.

It is well known that non-effective drugs and placebo pills will both show an average 30% improvement in depression scores from baseline, not just due to spontaneous improvement from waiting ^6^. This “placebo effect” is thought to be due to hope and expectation of improvement on the part of the patient ^7^.

We are also concerned that the term “evidence-based” is used in descriptions of the validity of a specific therapy without being clearly defined. While not foolproof, a double-blind design to control for expectations in antidepressant confirmatory studies is crucial in order to decrease potential bias ^2, 8^.

Non-experimental comparative designs may also be used to make clinical inferences, however, this requires that studies include a number of conditions including: that the study subjects need to provide valid observations for the biological question under study; and the effect of the treatment must be large compared with random error and bias ^2^. These conditions are extremely hard to meet in MDD where symptom reports are subjective ^1^.

We think that using the term “evidence-based” for both double-blinded clinical drug trials as well as for unblinded psychotherapy trials confuses a consistent definition for “evidence-based”. We opine that the most valid definition of “evidence-based” is that of evidence garnered from the results of confirmatory trials of antidepressants that require double-blinding ( http://cpnp.org/resource/mhc/2014/01/antidepressant-medications-fda-approval-process-and-need-updates) ^9^. In this way, clinical trial designs with the strongest control level would be the standard for “evidence-based” data, although we acknowledge that double-blind clinical trials may also have various design and/or operational problems leading to invalid results.

We would like to illustrate how the combination of the placebo effect, along with the inability to double-blind a psychotherapy trial, can lead to bias in the results.

Figure 1 illustrates the effect hope and expectation vs. pharmacological effect may have on improved depression scores. Ratings of depressive symptoms are subjective, some symptoms of which thus may be amenable to a subjective sense of improvement with the hope and/or expectation of entering a trial. Both subjects and ‘treaters’ are blind to the content of the pill received. Group B, is given a known antidepressant, but the subjects are blind to the nature of the pill. Blinding allows the study to show any unbiased antidepressant effect additional to hope and expectation.

**Figure 1.**
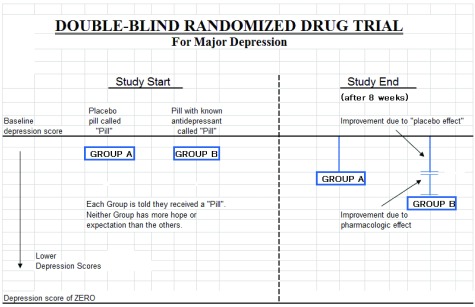
Double-blind randomized drug trial.

Even more than just saying a study was “blinded”, however, absolute concealment of what treatment was allocated is crucial in order to avoid bias ^10^. The study should clearly describe how they maintained the blind and employ an “exit analysis” to confirm that subjects were not aware of their treatment allocation.

Figure 2 illustrates a non-blinded psychotherapy efficacy trial, using a “discussion” group and a “CBT” group. Because subjects are openly receive the intervention given in a psychotherapy, it is essentially impossible to blind a psychotherapeutic intervention. Any type of psychotherapy could be used in this model, but CBT is an instructive case because there are workbooks and specific tasks given to subjects making it clear to subject that they are in the CBT group. The subjects and therapists are both told of the type of therapy received.

**Figure 2.**
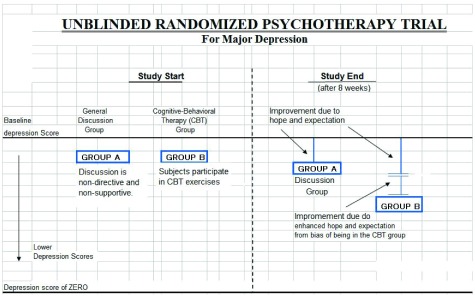
Unblinded randomized psychotherapy trial.

General Discussion refers to a non-directive, non-supportive discussion that is generally assumed to have no effect on MDD and represents a group that would have no expectation or hope of receiving a specific and directive therapy like CBT for the purposes of demonstrating the effect of unblinding for these groups. Assuming that “General Discussion” should not be effective in MDD, the discussion group’s improvement would then be similar to the placebo effect seen in a drug trial, and psychological placebos may also be as effective as accepted psychotherapies in MDD ^11^.

A third person who rates the degree of depression throughout the study should not know the therapy received (called a “masked rater”), but any bias on the part of the subject will just be part of the ratings reported by the subject to, and recorded by, the rater. The study is open so that it is not really possible to assess how much of each group’s improvement is due to the placebo effect, actual efficacy, or a bias towards CBT in knowing one is receiving CBT and/or hope from the act of actively trying to decrease negative thoughts as is done in CBT.

The importance of blinding in CBT interventions for psychiatric disorders was supported by a large meta analysis. Controlling for placebo and blindedness, a meta-analysis of data from published trials of CBT that showed CBT fared no better than non-specific control interventions in the treatment of schizophrenia and did not improve relapse rates, CBT showed no effect in prevention of bipolar disorder episodes, and only small treatment effects were seen in studies of MDD ^12^.

In our location in Japan, the Japanese National Health Insurance (NHI) system added CBT as a reimbursable procedure for MDD in 2010 ^13^. It is concerning to us for a National Health Insurance system to provide reimbursement for a treatment of MDD that does not have the same scientific rigor that a double-blind study of an antidepressant would have. This is similar to the NHI situation in the UK as discussed by Lynch ^12^.

The suicide rate in Japan is among the highest in the world ( http://www8.cao.go.jp/jisatsutaisaku/whitepaper/en/w-2013/pdf/chap1-1_p2-3.pdf, Accessed on December 4th, 2014.) ^14^, and it is possible that treatments for MDD that do not have confirmatory double-blind clinical trials may lead patients with serious depression away from other approved treatments that do have this confirmation. The fact that the organizational relationship of the Ministry of Health, Labour and Welfare (MHLW) who funded the CBT studies also decides on the make-up of the committees at an organization (called the Chuikyo) that determines approval for reimbursement by the NHI is also of concern ( http://www.japantimes.co.jp/life/2006/03/14/lifestyle/who-is-paying-the-price-of-health-care/#.VIAGhMlWrkc, Accessed March 20th, 2015) ^15^.

## Conclusions

The conclusions of the rationale presented in this paper would be that for MDD:

1. Pill placebos show considerable positive effect on disorders with subjective endpoints such as those used to rate MDD ^5– 7^.
2. Psychological placebos may be as effective as accepted psychotherapies ^11^.
3. Psychotherapy clinical trials are non-blinded studies, and cannot effectively be double-blinded. Calling these studies “single-blind” obfuscates the non-blinded nature of these studies and is not in line with the definition of “single-blind” in a clinical trial ^4^.
4. It is imperative that any intervention for a disorder with subjective endpoints such as MDD requires the same rigor in double-blinding in order to conclude that the results show “efficacy” or are “evidence-based”. This paper proposes to use the term, “partially-controlled clinical data” in place of “evidence-based clinical data” for results obtained from unblinded studies.

The implications for practice are, 1. For practitioners to be clear with patients the level of rigor to which interventions have been studied, 2. For authors of psychotherapy outcome studies to be clear that the problem in the inability to blind a psychotherapy trial severely restricts the validity of any conclusions that can be drawn, and 3. To petition National Health Insurance Plans to use caution in approving interventions studied without double-blinded confirmatory trials as they may lead patients to avoid other treatments shown to be effective in double-blinded trials.

The limitations of this paper are that the lack of double-blinding does also not prove that the psychotherapy intervention is not helpful in some way to the indication being treated. Clinical opinion and consensus may guide how a psychotherapy will be used in practice.

We hope this paper can stimulate more research related to problems in blinding of psychotherapy outcome studies, the potential economic and clinical costs of providing or not-providing private or national health reimbursement for psychotherapeutic interventions, and further discussion on how our official professional organizations and national research centers will define “evidence-based” in relation to interventions for major depressive disorder.
