# Supplementary material for: Double-Blinding and Bias in Medication and Cognitive-Behavioral Therapy Trials for Major Depressive Disorder
Source: F1000Res. 2016 Feb 4;4:638. Originally published 2015 Aug 27. [Version 2] doi: 10.12688/f1000research.6953.2 (PMC4732552; doi:10.12688/f1000research.6953.2)
Supplement: Peer review history

# Peer review: Referee response for version 1

**DOI:** 10.5256/f1000research.7488.r11959

**Published:** 2016-01-25

**Authors:** Keith R. Laws

**Affiliations:** 1 Department of Psychology, University of Hertfordshire, Hatfield, UK

I have a few comments on Dr Berger's manuscript, which raises interesting discussion points

1. Dr Berger states that “Psychotherapy clinical outcome trials for major depressive disorder (MDD) are often described as “randomized”, “controlled”, “singleblind”, etc. These words may not adequately describe the level of methodologic rigor of the design of a trial for MDD because *the endpoints are subjective symptom ratings*” (my italics).
  This latter point might benefit from some expanding and clarifying. All current psychiatric ratings are *subjective *to some extent (in pharma trials as well), but there are a variety of *subjectives. *For example, differences in terms of whether such 'subjective' assessment occurs using a self-rating tool (like the Beck Depression Inventory: BDI) or a clinician-rating scale (e.g. Hamilton Rating Scale for Depression). This is especially pertinent in depression trials as self-rating tools like BDI are frequently used and in effect, this renders trials 'open'.
  As Dr Berger states, meta-analyses show quite clearly that open psychotherapy trials can produce highly inflated effect sizes (see also Jauhar *et al*. 2014). The latter meta-analysis proves two points a) that compared with even *single-blind *trials, open trials of CBT can inflate effect sizes hugely and b) that contrary to what some say, psychotherapy trials can be very effectively blinded to assessors. 
2. I agree that the term ‘evidence-based’ in psychotherapy has been rendered somewhat meaningless and glosses over -what could be - important variability in terms of what this phrase covers. In some instances, paradoxically, the phrase is offered as an bland substitute for providing actual evidence...not unlike the often repeated “as recommended by NICE” - a shorthand way of avoiding the presentation and evaluation of actual evidence ... as if these phrases were chiselled in stone atop Mount Sinai.
3. Study quality is undoubtedly the key factor and conflating evidence from double-blind, single-blind and open trials under the rubric of ‘evidence-based’ is unhelpful - especially to patients who require guidance on the efficacy of prospective psychotherapy as Dr Berger argues. Indeed, it would be much more helpful to patients and clinicians if bodies such as NICE incorporate study quality *directly *into their analysis of the evidence-base and hence, their recommendations.
4. Its worth noting in passing that even when meta-analyses might reveal the same numerical effect size for a drug and a psychological intervention, this does not indicate *equivalence of efficacy*. Again, it depends crucially upon the quality of the data underpinning each effect size - so claims that CBT and anti-depressants have comparable effects really depends on the assumption that designs are equivalent (which they clearly are not). As Dr Berger suggests, drug trials are better controlled and so, any *equivalence *is more apparent than real.
5. The proposal to use “partially-controlled clinical data” in place of “evidence-based clinical data” certainly highlights a key difference, though I guess it all depends upon the extent to which psychological therapy can be squeezed into the 'RCT template' that has served drug evaluation so well…RCTs of psych therapies are *necessarily *at best single blind or at worst (and still very often) ‘open’. Some psychotherapy advocates would of course make a special case for psychotherapy - that it is simply not amenable to the same paradigms as drug studies and perhaps Dr Berger could expand on how he views the validity of such counter-arguments.

I have read this submission. I believe that I have an appropriate level of expertise to confirm that it is of an acceptable scientific standard.

## References

[1] JauharSMcKennaPJRaduaJFungESalvadorRLawsKR: Cognitive-behavioural therapy for the symptoms of schizophrenia: systematic review and meta-analysis with examination of potential bias. *Br J Psychiatry*.2014;204(1) : 10.1192/bjp.bp.112.116285 20-9 10.1192/bjp.bp.112.116285 24385461

# Peer review: Referee response for version 1

**DOI:** 10.5256/f1000research.7488.r10898

**Published:** 2015-10-23

**Authors:** Gordon Parker

**Affiliations:** 1 School of Psychiatry, University of New South Wales, Sydney, NSW, Australia

A straightforward paper, well written and taking the reader through the difficulties of undertaking and interpreting psychotherapies when no placebo control or equivalent control strategy is available and therefore worthy of indexing.

I have read this submission. I believe that I have an appropriate level of expertise to confirm that it is of an acceptable scientific standard.
